# A Case Report of Laparoscopic Left-Sided Appendectomy in a Child With Situs Inversus

**DOI:** 10.7759/cureus.24426

**Published:** 2022-04-23

**Authors:** Essa A Khudhayr, Abdullah A Ali, Ehab Alameer

**Affiliations:** 1 General Surgery, Armed Forces Hospital Southern Region, Khamis Mushait, SAU; 2 Surgery, Faculty of Medicine, Jazan University, Jazan, SAU

**Keywords:** case report, laparoscopic appendectomy, left lower quadrant, appendicitis, situs inversus

## Abstract

Acute appendicitis classically elicits right-sided abdominal pain. Abdominal pain in patients with situs inversus may create a diagnostic challenge. We share a case of a seven-year-old boy who presented to the emergency department with left lower quadrant abdominal pain and tenderness. A chest x-ray showed dextrocardia and an abdominal ultrasound confirmed left-sided appendicitis. Diagnostic laparoscopy and appendectomy were uneventful. Thorough peri-operative evaluation and planning are key to a successful outcome in this rare condition

## Introduction

Appendicitis is a common surgical emergency, and one of the most common causes of acute abdominal pain. Reported incidence rate is 4-8% [1-3]. The classic presentation is acute peri-umbilical pain that eventually localizes to the right lower quadrant [1-3]. About one-third of patients diagnosed with acute appendicitis experience abdominal pain in atypical sites due to variation in the anatomical location of the appendix (e.g., pelvic, pre-ileal, post-ileal, retrocecal, or sub-cecal areas). The appendiceal tip may be found in even more rare sites (e.g., mesocolic, subhepatic, right side long appendix reaching up to the left lower quadrant of the abdomen) potentially creating a diagnostic confusion [2,3]. Diagnosis of acute appendicitis is made based on history and physical symptoms. Imaging is not always necessary but may be helpful when in doubt [1,3].

The rarity of left side acute appendicitis may lead to misdiagnosis. Left lower quadrant pain could be attributed to any of many differential diagnoses [2-4]. Some of these diagnoses include gastrointestinal etiologies, such as diverticular disease, mesenteric ischemia, bowel obstruction, left-sided primary epiploic appendagitis, and genitourinary diseases such as ectopic pregnancy, ovarian torsion, pelvic inflammatory disease, cystitis, epididymitis, prostatitis, and testicular torsion [1-4]. Left acute appendicitis is predisposed by two congenital conditions: situs inversus and congenital malrotation of the midgut [4-6]. In the current case, we discuss a case of left acute appendicitis in a child with situs inversus, which was successfully managed through a laparoscopic approach.

## Case presentation

**Figure 1:**
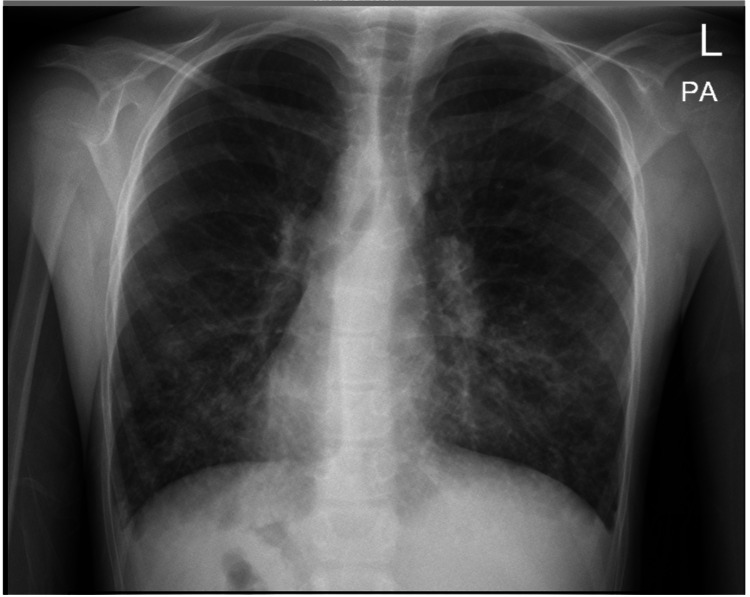
Chest x-ray (posterior anterior view) showing dextrocardia

A seven-year-old boy, known to have Kartagener syndrome, presented to the ED with complaints of left lower abdominal pain and vomiting for one day. He had no fever, change in bowel habits, or urinary symptoms. On physical examination, tenderness was evident in the left iliac fossa with positive rebound tenderness. Lab data was significant for leukocytosis of 23,000 with neutrophilia. A chest x-ray showed dextrocardia (Figure 1).

**Figure 2:**
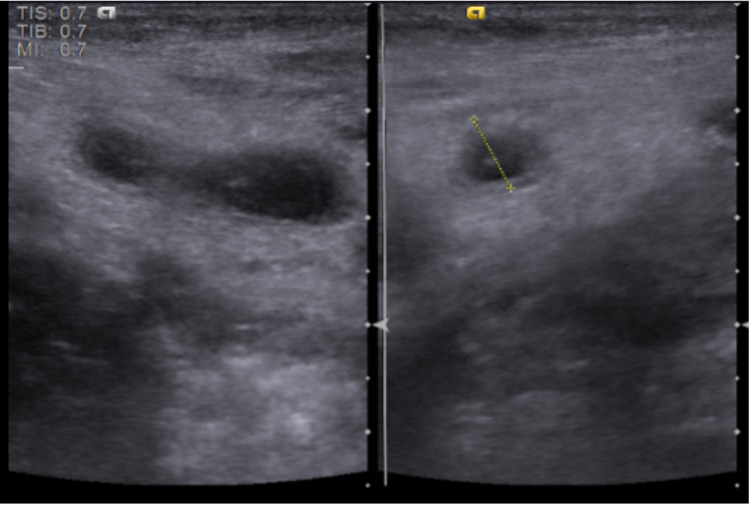
Abdominal ultrasound was consistent with inflamed left-sided appendix

**Figure 3:**
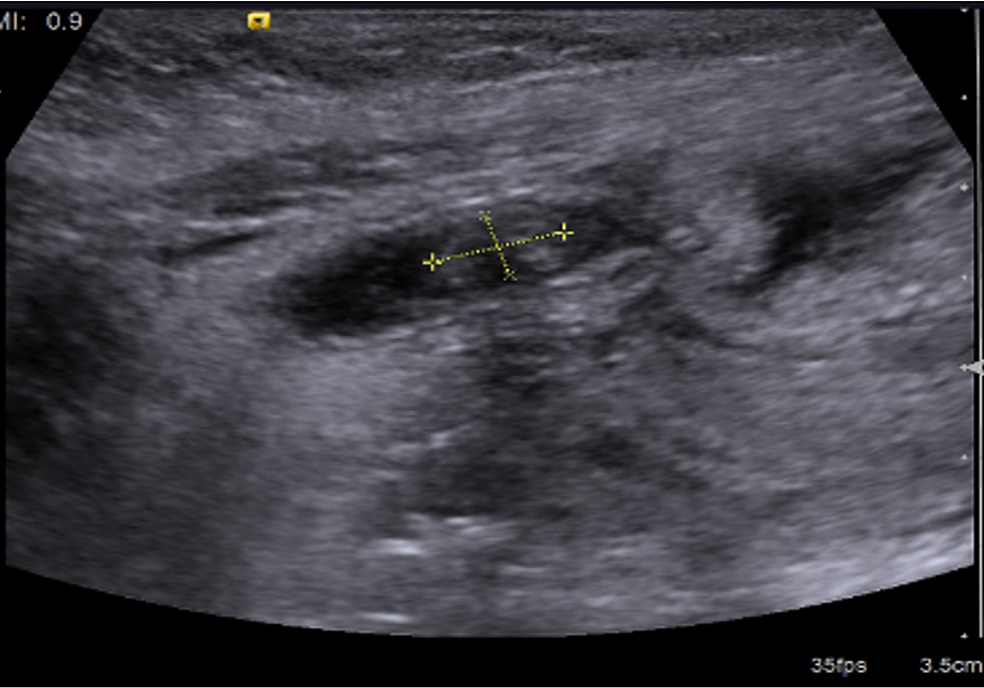
Abdominal ultrasound was consistent with inflamed left-sided appendix

An abdominal ultrasound (USG) revealed the liver in the left side of the abdomen and the spleen on the opposite side. A blind-ended mildly thickened-walled, distended tubular structure was seen at the left iliac/lumbar region, measuring about 7 mm at its mid-portion transverse diameter (Figure 2), eliciting internal inhomogeneous fluid contents; appendicolith was also seen near its origin measuring about 6.5 x 3.5 mm at its maximum axial diameter (Figure 3). This was associated with peri-appendiceal inflamed, thickened, and edematous bright mesenteric fatty thickening, as well as minimal peri-appendiceal reactive inflammatory fluid streaks. The appendix appeared to arise from the caecum at the left iliac fossa and, coursing cranially, reach the lumbar region.

**Figure 4:**
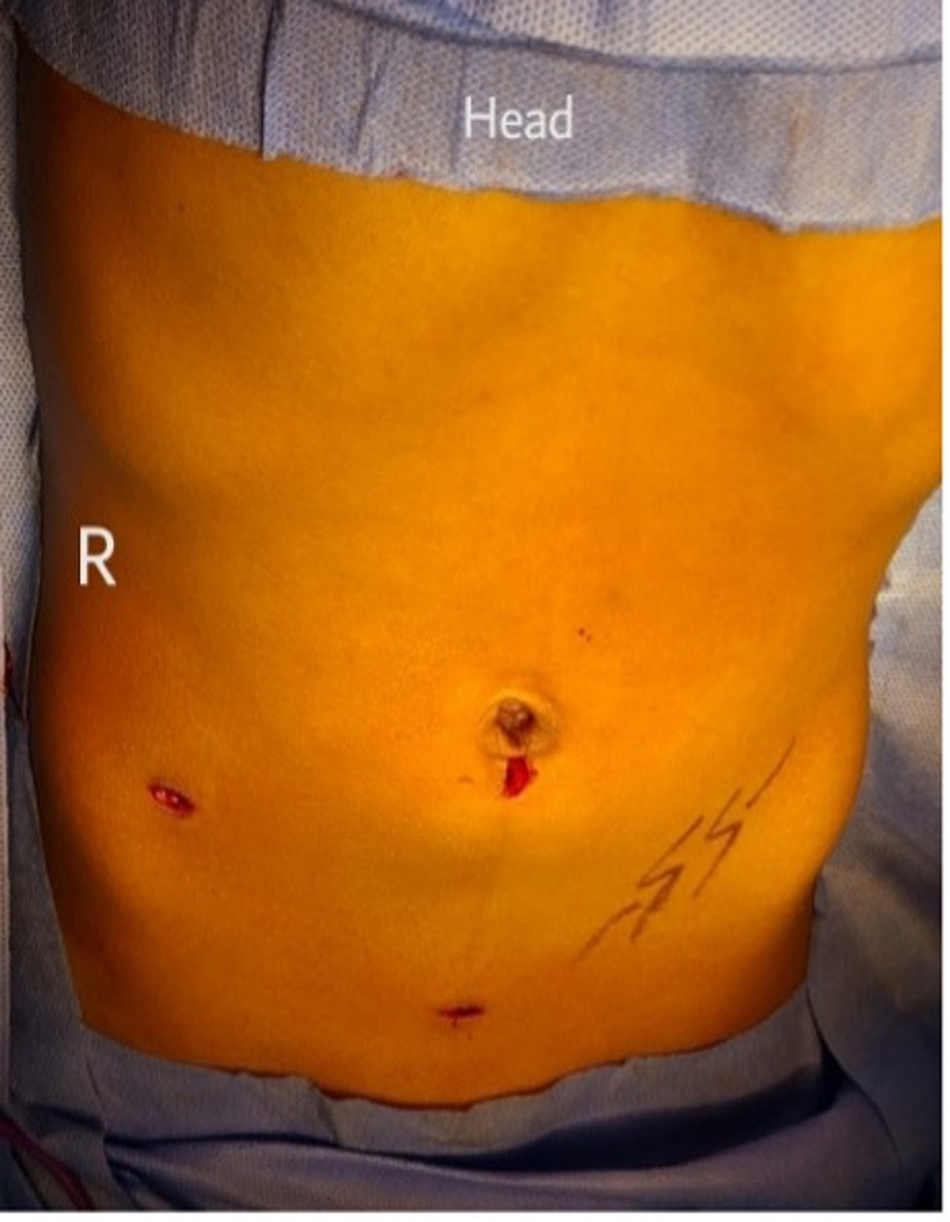
laparoscopic ports sites

**Figure 5:**
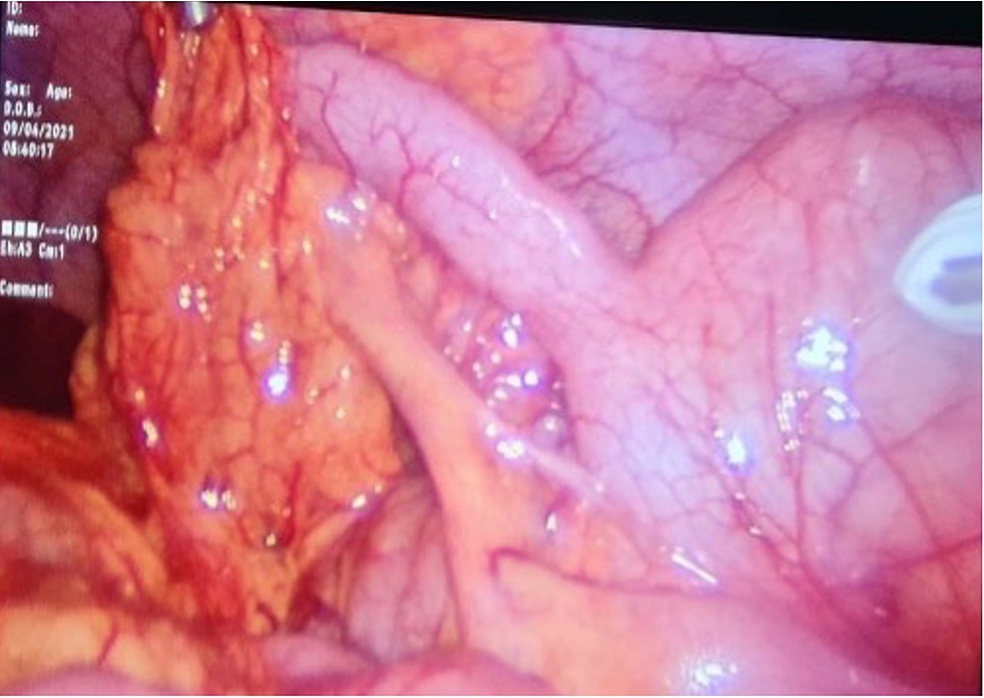
Laparoscopic view of the appendix showing omental adhesions and left-sided caecum

Diagnostic laparoscopy and appendectomy were performed. Operating room setup and trocars placement were modified in a “mirror image” of the standard laparoscopic appendectomy. Pneumoperitoneum was induced using the Veress needle technique, followed by the insertion of a 5 mm port at the umbilicus (Figure 4). The telescope confirmed a left-sided, thick inflamed appendix, coursing cranially to the lumbar region (Figure 5). A 12 mm port was placed in the right iliac fossa (RIF), and a 5 mm port was placed in the suprapubic region, both under direct visualization. The appendectomy was uneventful and the patient recovered well.

## Discussion

Situs inversus is a rare congenital anomaly [2,3,7,8]. Defect is localized on the long arm chromosome 14 [3,8]. It is autosomal recessive disease characterized by transposition of the organs inside the chest and abdomen [1,2,4,5,7,8]. The incidence of situs inversus ranges between 0.001% and 0.01% in general population [1,4,5,7]. The incidence of acute appendicitis in patients with situs inversus is exceedingly low (0.016% to 0.024%) [1,4,6]. During embryogenesis, abdominal organs normally turn 270 counterclockwise, which results in the appendix been in the right side of the abdomen [7,8]. Situs inversus happens when the rotation is 270 clockwise, causing a left-sided appendix [7,8].

Left-sided acute appendicitis can pose a diagnostic challenge and thus requires a high index of suspension. The correct diagnosis could be reached based on history taking, physical examination, imaging studies (chest x-ray, USG, CT), and diagnostic laparoscopy [1,7]. Although a plain x-ray is not useful for diagnosing appendicitis, dextrocardia can be detected in a chest x-ray, which can lead to diagnosing situs inversus. Moreover, a right-sided gastric gas bubble can be observed on an abdominal plain x-ray [2,4,6,7]. In the last two decades, acute appendicitis has mostly been diagnosed by USG and CT scans [6]. USG is most commonly used. It is operator-dependent, and some findings can be obscured with large body habitus or overlying bowel gases [6,7]. CT scan is more precise than USG and has a reported diagnostic accuracy rate of 90-98% for acute appendicitis. It can provide a confirmation of situs inversus in addition to detecting acute appendicitis in this group of patients [2,5-7].

Laparoscopic appendectomy is a safe and efficient approach for treating acute appendicitis. This technique has several advantages over open laparotomy. It has been associated with quicker recovery, less post-operative hospital stay, less psychological stress, and fewer postoperative complications [9,10]. Laparoscopy has the additional benefit of confirming situs inversus if not diagnosed preoperatively, and it enables the surgeon to examine intra-abdominal structures in case of any diagnostic uncertainty. As described earlier, a few adjustments of the operating room setup and trocars placement are necessary for a successful surgery. The surgeon and the assistant stand on the patient’s right side and the monitor is placed on the left side of the patient. The reverse laparoscopic view can be a technical challenge for the non-experienced surgeon [3] and a right-handed surgeon may have to dissect using the left hand. In contrast to the standard procedure, there is no standard position for the insertion of trocars in these particular cases, and port placement should be modified by the surgeon following laparoscopic surgery principles [11,12]. Post-operative care is not different from standard laparoscopic appendectomy. Patient and family should be educated about the implications of situs inversus and the potential for future diagnostic confusion.

## Conclusions

Acute appendicitis in patients with situs inversus can be challenging. High index of suspicion and thorough clinical evaluation are paramount. Diagnostic laparoscopy and appendectomy are recommended. Adjustment of operating room setup and trocars placement may facilitate surgery. Clinicians should be aware of rare anatomic variations and their implications.
